# Developmental Patterns and Risk Factors of Scoliosis After Hemipelvectomy for the Pelvic Bone Tumor

**DOI:** 10.3390/diagnostics14212392

**Published:** 2024-10-27

**Authors:** Ryuto Tsuchiya, Shintaro Iwata, Suguru Fukushima, Shuhei Osaki, Koichi Ogura, Eisuke Kobayashi, Seiji Ohtori, Akira Kawai

**Affiliations:** 1 Department of Musculoskeletal Oncology and Rehabilitation, National Cancer Center Hospital, 5-1-1 Tsukiji, Chuo-ku, Tokyo 104-0045, Japan; ryuto19890713@gmail.com (R.T.); fsgru0428@gmail.com (S.F.); sosaki@ncc.go.jp (S.O.); koogura@ncc.go.jp (K.O.); ekobayas@ncc.go.jp (E.K.); akawai@ncc.go.jp (A.K.); 2 Department of Orthopaedic Surgery, Graduate School of Medicine, Chiba University, 1-8-1 Inohana, Chuo-ku, Chiba 260-8670, Japan; sohtori@faculty.chiba-u.jp

**Keywords:** hemipelvectomy, scoliosis, sarcoma

## Abstract

Background: Postoperative scoliosis is often seen after hemipelvectomy for malignancies involving the pelvic area, but the details remain unclear. The objectives were to investigate the development patterns and risk factors of scoliosis after hemipelvectomy. Methods: We retrospectively reviewed 30 patients who underwent hemipelvectomy at our hospital between 1998 and 2020. The risk factors of scoliosis with a Cobb angle of ≥10° were investigated. Results: The postoperative Cobb angle significantly increased in all patients compared with the preoperative one (*p* < 0.001), and the change ratio of the Cobb angle was significantly higher during the first postoperative year than thereafter. The external hemipelvectomy (EH) group demonstrated a larger Cobb angle and a higher change ratio than the internal hemipelvectomy group. Nine patients developed scoliosis with a final Cobb angle of ≥10°, and the risk factors were EH (*p* = 0.017), P1+2+3+4 resection according to the Enneking classification (*p* = 0.005), iliac crest resection (*p* = 0.004), L5/S resection (*p* = 0.020), and no pelvic ring reconstruction after hemipelvectomy (*p* = 0.004). Conclusions: Approximately 30% of patients who underwent hemipelvectomy developed scoliosis with a Cobb angle of ≥10°, and this angle increased rapidly during the first postoperative year. Hence, careful follow-up of scoliosis is required after hemipelvectomy.

## 1. Introduction

Hemipelvectomy is a definitive surgery for malignant bone and soft tissue tumors involving the pelvic area [1,2,3]. It consists of two methods: external hemipelvectomy (EH; a combined resection of the lower extremity) and internal hemipelvectomy (IH; preservation of the lower extremity) [4,5,6,7]. The choice between EH or IH depends on the extent of tumor advancement. Generally, IH is difficult to perform when the tumor already involves critical blood vessels or nerves. However, recent advances in adjuvant therapy, such as preoperative chemotherapy or radiotherapy, have made limb-sparing surgery more feasible [1,3,6,7,8]. Various reconstruction methods have also been recently developed; these methods include megaprosthesis, massive allograft, autograft/recycling bone, arthrodesis, and hip transposition [9,10,11,12].

Although hemipelvectomy is a curative treatment modality, it is one of the most challenging surgeries for the musculoskeletal surgeon because of the complex neurovascular components and abdominal viscera around the pelvis [1]. Additionally, the surgical invasion is extremely high, leading to a higher complication rate compared with other surgical procedures. Infection, vessel injury, nerve injury, and wound healing disorder are well-known postoperative complications of hemipelvectomy [1,6,7,8,13,14,15].

A few studies reported scoliosis as another complication after hemipelvectomy [4,10,16]. Generally, spinal deformity with a Cobb angle of ≥10 is defined as degenerative scoliosis [17,18]. In patients with a Cobb angle of ≥30 accompanied with severe low back pain and neurological symptoms, surgical treatment is considered [18,19]. Therefore, postoperative scoliosis is a crucial complication that requires attention. However, to our best knowledge, no detailed study has examined the development patterns or risk factors of scoliosis after hemipelvectomy. Therefore, we conducted a retrospective observational study to examine the scoliosis after hemipelvectomy.

## 2. Materials and Methods

### 2.1. Study Design and Setting

Patients were included in this study according to the following criteria: (1) they underwent hemipelvectomy at our hospital between 1998 and 2020, (2) they had survived at least 1 year after surgery, (3) they were regularly followed up by CT, and (4) they had a disrupted pelvic ring. Patients who received partial pelvic resection without pelvic ring disruption were excluded because this procedure has little effect on pelvic shape. Thus, 30 patients (13 males and 17 females) were included, with a median age of 47.5 years (interquartile range [IQR], 25.8–64.3 years) and a median follow-up time of 60.0 years (IQR, 30.7–98.9 years). We found 13 patients who received EH and 17 who received IH. The area of pelvic resection according to the Enneking classification [20] was P2, P1+2, P2+3, P1+2+3, and P1+2+3+4 in 2, 4, 8, 10, and 6 patients, respectively. Of the 13 patients who received EH, 2 underwent pelvic ring reconstruction with fibula grafts. In the IH group, 11 underwent hip transposition, and 6 underwent reconstruction with megaprosthesis. Histologically, 10, 8, 2, 2, and 8 patients were diagnosed with osteosarcoma, chondrosarcoma, undifferentiated pleomorphic sarcoma, malignant peripheral nerve sheath tumor, and other tumors, respectively. Table 1 and Table S1 summarize the clinical information of these 30 patients. Furthermore, the institutional review board of our institute approved this study.

### 2.2. Parameter Measurements

Based on the CT coronal view, the Cobb angle was measured using CT images taken preoperatively and at 1, 2, and 3 years postoperatively, and each time point was defined as Pre, Post1, Post2, and Post3, respectively. The Cobb angle was measured twice, and the mean value was determined. The change ratio of the Cobb angle between Post1–Pre, Post2–Post1, and Post3–Post2 was also calculated, and each interval was defined as 1st, 2nd, and 3rd intervals, respectively. The curve level and direction were also evaluated. When the patients had a double curve, the caudal scoliosis curve was used in the analysis. Moreover, we examined whether the resection area included L5/S and whether the iliac crest was resected or not.

### 2.3. Evaluation of the Risk Factors of Scoliosis Development After Hemipelvectomy

In this study, a Cobb angle of ≥10° indicated scoliosis, and the risk factors of scoliosis development after hemipelvectomy were examined. To determine the presence of statistically significant factors, we conducted the Mann–Whitney U test for continuous variables, and the chi-square test or Fisher’s exact test for categorical variables. GraphPad Prism 8.4.1 software (GraphPad Inc., San Diego, CA, USA) was used for these procedures. We considered a *p* value of <0.05 statistically significant.

## 3. Results

### 3.1. Time-Course Change of Cobb Angle in All Patients

**Figure 1:**
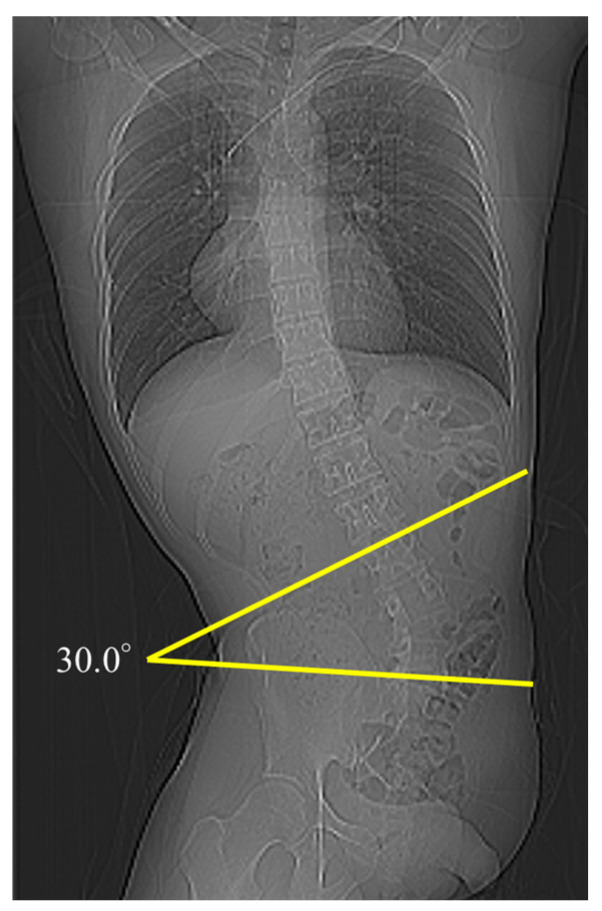
CT scout image of a patient who had undergone hemipelvectomy.

**Figure 2:**
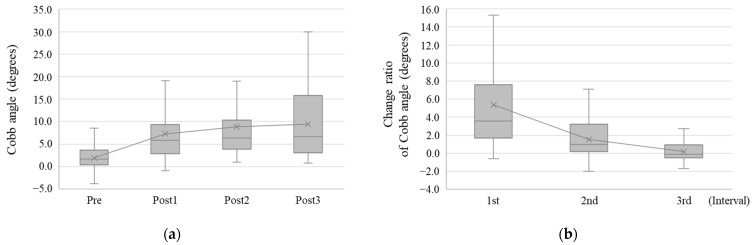
Cobb angle change of all patients. (**a**) Time-course change of the Cobb angle. The Cobb angle increased over time after hemipelvectomy. (**b**) The change ratio of the Cobb angle. The change ratio was prominent during the first year after hemipelvectomy. In the x-axis, Pre means the preoperative time point, and PostX means X years after hemipelvectomy. The intervals between Post1–Pre, Post2–Post1, and Post3–Post2 were described as 1st, 2nd, and 3rd intervals, respectively.

We observed 30, 30, 25, and 20 patients at Pre, Post1, Post2, and Post3, respectively. At final observation, nine (30%) patients developed scoliosis with a Cobb angle of ≥10°. Among these nine patients, one developed scoliosis with a Cobb angle of 20°–30°, and one developed severe scoliosis with a Cobb angle of ≥30° (Figure 1). The median Cobb angle at Pre, Post1, Post2, and Post3 was 1.7° (IQR, 0.4°–3.7°), 5.9° (IQR, 2.8°–9.4°), 6.4° (IQR, 3.9°–10.3°), and 6.7° (IQR, 3.1°–15.9°), respectively. Therefore, the postoperative Cobb angle was significantly increased compared with the preoperative one in all patients (*p* < 0.001) (Figure 2a).

In addition, the change ratio of Cobb angle at 1st, 2nd, and 3rd intervals was 3.6° (IQR, 1.7°–7.6°), 1.0° (IQR, 0.2°–3.2°), and −0.1° (IQR, −0.5° to 1.0°), respectively (Figure 2b). Thus, the change ratio of the Cobb angle was significantly the highest during the 1st interval (*p* < 0.001).

### 3.2. Effect of EH and IH on the Change of Cobb Angle

**Figure 3:**
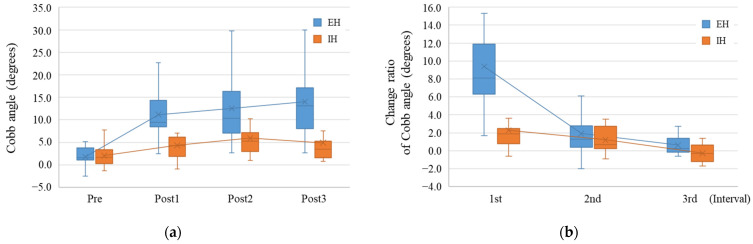
Cobb angle change in the external hemipelvectomy group and internal hemipelvectomy group. (**a**) Time-course change of the Cobb angle in the external hemipelvectomy (EH, blue box plot) group and internal hemipelvectomy (IH, orange box plot) group. The Cobb angle in the EH group was significantly larger than that in the IH group. (**b**) The change ratio of the Cobb angle in each group. The change ratio was higher in the EH group than in the IH group during the first year after surgery. In the x-axis, Pre means the preoperative time point, and PostX means X years after hemipelvectomy.

We examined and compared the Cobb angle and its change ratio between the EH and IH groups. The median Cobb angle at Pre, Post1, Post2, and Post3 was 1.6°, 9.4°, 10.3°, and 13.2° in the EH group and 1.7°, 4.3°, 5.3°, and 3.5° in the IH group, respectively (Figure 3a). Although the Cobb angle at Pre was not significantly different between the two groups (*p* = 0.992), the Cobb angles at Post1, Post2, and Post3 were significantly larger in the EH group than in the IH group (*p* < 0.001, *p* = 0.015, and *p* = 0.007, respectively).

Moreover, the median change ratio at the 1st, 2nd, and 3rd intervals was 8.1°, 1.3°, and 0.3° in the EH group and 1.9°, 0.7°, and −0.3° in the IH group, respectively (Figure 3b). The change ratio at the 1st interval was significantly higher in the EH group than in the IH group (*p* < 0.001). Conversely, the change ratio at the 2nd and 3rd intervals was not very different between the two groups (2nd interval, *p* = 0.491; 3rd interval, *p* = 0.105).

### 3.3. Effect of L5/S Resection on the Change of Cobb Angle

**Figure 4:**
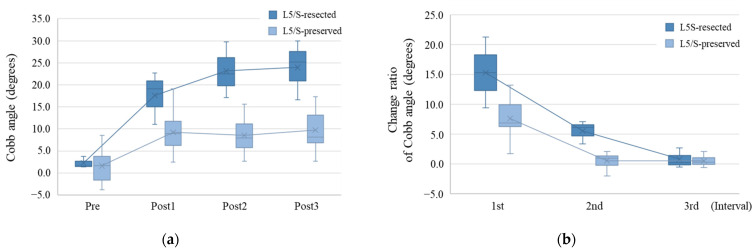
Effect of L5/S resection on Cobb angle change in the external hemipelvectomy group. (**a**) Time-course change of the Cobb angle in the L5/S-resected group (deep blue box plot) and L5/S-preserved group (light blue box plot) among the EH groups. The Cobb angle was significantly larger in the L5/S-resected group than in the L5/S-preserved group. (**b**) The change ratio of the Cobb angle in each group. The change ratio was higher in the L5/S-resected group than in the L5/S-preserved group between the 1st and 2nd intervals. In the x-axis, Pre means the preoperative time point, and PostX means X years after hemipelvectomy.

We further divided the EH group, which showed prominent Cobb angle change, into two groups: the L5/S-resected group (*n* = 3) and the L5/S-preserved group (*n* = 10). The median Cobb angles at Pre, Post1, Post2, and Post3 were 1.6°, 19.1°, 22.5°, and 25.2° in the L5/S-resected group and 1.7°, 8.9°, 8.0°, and 8.1° in the L5/S-preserved group, respectively (Figure 4a). Therefore, the Cobb angle at Post1 tended to be larger in the L5/S-resected group than in the L5/S-preserved group (*p* = 0.052), and those at Post2 and Post3 were significantly larger in the L5/S-resected group (*p* = 0.012 and *p* = 0.033, respectively).

In addition, the median change ratios at the 1st, 2nd, and 3rd intervals were 15.3°, 6.1°, and 0.2° in the L5/S-resected group and 6.9°, 1.0°, and 0.3° in the L5/S-preserved group (Figure 4b). Thus, the L5/S-resected group had significantly higher change ratios at the 1st and 2nd intervals than the L5/S-preserved group (*p* = 0.046 and *p* = 0.012, respectively). At the 3rd interval, the change ratio was not significantly different between the two groups (*p* > 0.999).

### 3.4. Factors That Affected the Curve Direction of Scoliosis

**Figure 5:**
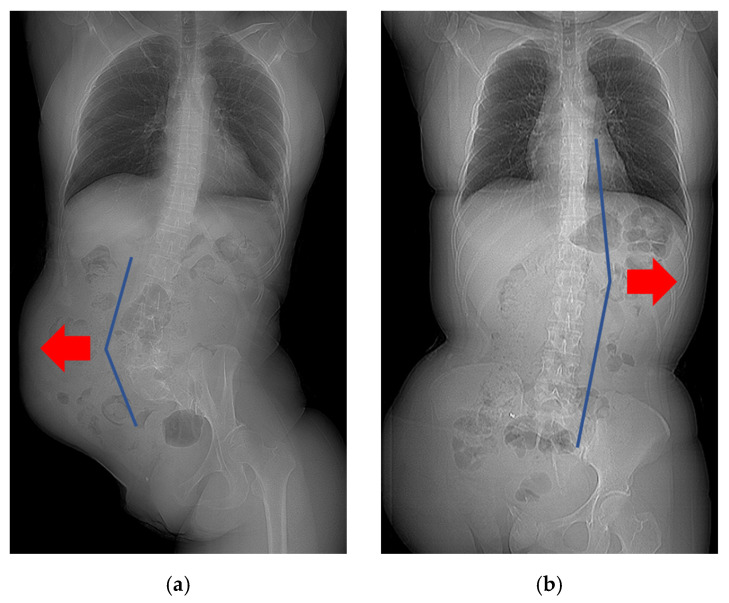
Relationship between iliac crest resection and the curve direction of scoliosis. (**a**) A patient who underwent EH with iliac crest resection demonstrated a convex curve toward the ipsilateral side of the resection. (**b**) A patient who underwent EH with iliac crest preservation demonstrated a convex curve toward the contralateral side of the resection. Red arrows indicate the convex side.

In patients who underwent EH with iliac crest resection (*n* = 9), seven demonstrated a convex curve toward the ipsilateral side of the resection. Conversely, those with iliac crest preservation (*n* = 4) demonstrated a convex curve toward the contralateral side of the resection (Figure 5, Table S1). Thus, the side of iliac crest resection was related to the curve direction of scoliosis in the EH group (*p* = 0.021). In the IH group, the curve direction was variable.

### 3.5. Risk Factors of Scoliosis After Hemipelvectomy

We examined the risk factors of scoliosis development with a Cobb angle of ≥10°. In the univariate analysis, the risk factors of scoliosis were EH (*p* = 0.017), P1+2+3+4 resection (*p* = 0.005), iliac crest resection (*p* = 0.004), L5/S resection (*p* = 0.020), and no reconstruction after hemipelvectomy (*p* = 0.004) (Table 2).

## 4. Discussion

Hemipelvectomy is a definitive surgery for malignant bone and soft tissue tumors involving the pelvic area [1,2,3]. Although postoperative scoliosis has been reported as one of its complications [4,10,16], the reports are still limited. In addition, the development patterns or risk factors of scoliosis after hemipelvectomy remain unclear. Hence, we examined scoliosis after hemipelvectomy.

After hemipelvectomy, the Cobb angle increased rapidly during the first year and gradually increased thereafter. This trend was particularly prominent in the EH group. Cases of rapid scoliosis development with a Cobb angle of ≥30° after EH were reported previously [16]. In our study, the Cobb angle increased, albeit slowly, after the first year. The rapid progression of scoliosis in the first year may be a compensation in response to sudden structural changes such as leg length discrepancy and pelvic tilt angle following hemipelvectomy [21,22]. Therefore, it is assumed that after the body balance was improved through compensation by the scoliosis, the progression slowed down. Moreover, hemipelvectomy has a 5-year overall survival rate of 40–45%; thus, its clinical postoperative outcome is considered unfavorable. Nevertheless, owing to the improved outcomes of chemotherapy and radiation therapy, the number of patients experiencing long-term survival after hemipelvectomy may increase [1,5,8]. Therefore, a continuous follow-up of scoliosis progression is required not only during the first postoperative year but also thereafter.

Prominent scoliosis progression was observed in the EH group with L5/S resection, a characteristic feature of scoliosis progression after hemipelvectomy. The intervertebral disc, iliolumbar ligament, and paraspinal muscles in the pelvis–spine region contribute to spinal stability [23,24,25,26]. Therefore, patients who received EH, especially those with L5/S disruption caused by the extended resection, exhibited rapid progression of scoliosis. Conversely, the IH group showed a slower increase of Cobb angle than the EH group, possibly because the IH group underwent hip transposition or megaprosthesis reconstruction, resulting in the compensation of leg-length discrepancy. However, the leg-length discrepancy is inevitable to a certain extent even after IH, and prolonged leg-length discrepancy can cause structural scoliosis [21,22]. Although scoliosis slowly progresses in the IH group, one patient with scoliosis and lumbar disk herniation reportedly required surgical treatment 6 years after IH [10].

We also found that iliac crest resection was related to the curve direction of scoliosis. The quadratus lumborum muscle, which is attached to the iliac crest, is responsible for maintaining the equilibrium of pelvic obliquity in the coronal plane [24]. Therefore, pelvic obliquity caused by iliac crest resection may affect the curve direction of scoliosis. Radiculopathy generally occurs on the concave side of scoliosis; thus, it might affect the residual lower extremity of the EH group, which tends to have a convex scoliosis toward the resected side [27,28]. When radiculopathy occurred on the residual lower extremity in patients who underwent EH, the functional outcome would worsen, requiring careful observation.

In this study, nine (30%) patients had a Cobb angle of ≥10°, and the risk factors of scoliosis development were P1+2+3+4 resection, iliac crest resection, L5/S area resection, and no post-hemipelvectomy reconstruction. According to the results, an extensive resection would be critical for scoliosis development postoperatively. Although the resection area depends on the extent of tumor spread, we should pay more attention to the development of scoliosis in patients with iliac crest and L5/S area resection. In addition, patients who did not receive any reconstruction have a risk of developing scoliosis postoperatively. Of note, all of these patients underwent EH; thus, a selection bias is possible. Nonetheless, scoliosis did not occur in two patients who underwent EH and pelvic ring reconstruction with fibula grafts. Pelvic ring reconstruction could lead to better functional results after hemipelvectomy [10,11], and it might also prevent scoliosis. Furthermore, none of the patients who underwent IH and reconstruction with megaprosthesis showed scoliosis. In contrast, scoliosis occurred in 18.2% of the patients who underwent IH and reconstruction with hip transposition. Postoperative functional outcomes after prosthetic reconstruction and hip transposition are still under discussion [29,30,31]. Considering the risk of scoliosis, prosthetic reconstruction may be more favorable than hip transposition, possibly because of less leg-length discrepancy.

This study is, to our best knowledge, the first to examine scoliosis after hemipelvectomy in detail. However, it has several limitations. First, the number of patients included in this study is relatively small. Therefore, multivariate analysis could not be conducted, and the possibility of interactions cannot be ruled out. However, considering the limited number of patients for whom hemipelvectomy is indicated and their prognosis, we believe that the cohort used in this study is valuable and significant. Second, this study is based on CT imaging, not on whole-spine standing X-ray. The supine position reportedly underestimates the Cobb angle by 5°–10° compared with the standing position, and the actual Cobb angle may be considerably larger [32,33]. Consequently, the actual incidence of scoliosis after hemipelvectomy may be higher than that in the present study. Third, owing to its retrospective study design, we could not investigate neurological symptoms and the low back pain accompanied with scoliosis. Finally, we could not confirm whether or not prosthetic legs were used after the operation. Although there are such limitations, scoliosis after hemipelvectomy is highly significant as it could directly affect the patient’s activities of daily living. With advancements in chemotherapy and radiotherapy improving patient prognosis, selecting surgical techniques that are less likely to result in scoliosis after hemipelvectomy may offer functional advantages in the future. However, since the number of patients eligible for hemipelvectomy is limited, it will be necessary to accumulate cases through multi-institutional collaboration for more detailed investigation.

## 5. Conclusions

In conclusion, 30% of all included patients who underwent hemipelvectomy developed scoliosis with a Cobb angle of ≥10°, and multiple risk factors have been identified. In particular, the EH group, especially those with L5/S resection, developed severe scoliosis, and the Cobb angle increased rapidly during the first year after hemipelvectomy. In addition, the iliac crest resection contributed to the curve direction of scoliosis. Radiculopathy and severe low back pain caused by post-hemipelvectomy scoliosis may be related to the poor functional prognosis. Thus, postoperative scoliosis should be carefully followed up.

## Figures and Tables

**Table 1:** Patient characteristics.

	EH Group (*n* = 13)	IH Group (*n* = 17)	Overall (*n* = 30)
Age (years), median [IQR]	49 [25–66]	46 [28–59]	47.5 [25.8–64.3]
Sex			
Male	4 (30.8%)	9 (52.9%)	13 (43.3%)
Female	9 (69.2%)	8 (47.1%)	17 (56.7%)
Enneking classification			
P2	0 (0%)	2 (11.8%)	2 (6.7%)
P1+2	1 (7.7%)	3 (17.6%)	4 (13.3%)
P2+3	3 (23.1%)	5 (29.4%)	9 (30%)
P1+2+3	4 (30.8%)	6 (35.3%)	9 (30%)
P1+2+3+4	5 (38.5%)	1 (5.9%)	6 (20%)
Iliac crest resection			
Yes	9 (69.2%)	9 (52.9%)	18 (60%)
No	4 (30.8%)	8 (47.1%)	12 (40%)
L5/S resection			
Yes	3 (25%)	0 (0%)	3 (10%)
No	10 (75%)	17 (100%)	27 (90%)
Reconstruction			
No reconstruction	11 (84.6%)	-	11 (36.7%)
Pelvic ring reconstruction	2 (15.4%)	-	2 (6.7%)
Hip transposition	-	11 (64.7%)	11 (36.7%)
Megaprosthesis	-	6 (35.3%)	6 (20%)
Diagnosis			
Osteosarcoma	5 (38.5%)	5 (29.4%)	10 (33.3%)
Chondrosarcoma	3 (25%)	5 (29.4%)	8 (26.7%)
Undifferentiated pleomorphic sarcoma	2 (15.4%)	0 (0%)	2 (6.7%)
Malignant peripheral nerve sheath tumor	1 (7.7%)	1 (5.9%)	2 (6.7%)
Others	2 (15.4%)	6 (35.3%)	8 (26.7%)

EH, external hemipelvectomy; IH, internal hemipelvectomy; IQR, interquartile range.

**Table 2:** Risk factors of scoliosis after hemipelvectomy.

	*n*	Scoliosis (+)	Scoliosis (−)	*p* Value
Age (years), median [IQR]		49 [25–66]	46 [28–67]	>0.999
Sex				0.229
Male	13	2 (15.4%)	11 (84.6%)	
Female	17	7 (41.2%)	10 (58.8%)	
Surgical procedure				0.017
External hemipelvectomy	13	7 (53.8%)	6 (46.2%)	
Internal hemipelvectomy	17	2 (11.8%)	15 (88.2%)	
Enneking classification				0.005
P1+2+3+4	6	5 (83.3%)	1 (16.7%)	
Others	24	4 (16.7%)	20 (83.3%)	
Iliac crest resection				0.004
Yes	18	9 (50%)	9 (50%)	
No	12	0 (0%)	12 (100%)	
L5/S resection				0.020
Yes	3	3 (100%)	0 (0%)	
No	27	6 (22.2%)	21 (77.8%)	
Reconstruction				0.004
No reconstruction	11	7 (63.6%)	4 (36.4%)	
Reconstruction group (Pelvic ring reconstruction + Hip transposition + Megaprosthesis)	19	2 (10.5%)	17 (89.5%)	
Diagnosis				0.300
Osteosarcoma	10	4 (40%)	6 (60%)	
Chondrosarcoma	8	4 (50%)	4 (50%)	
Undifferentiated pleomorphic sarcoma	2	0 (0%)	2 (100%)	
Malignant peripheral nerve sheath tumor	2	0 (0%)	2 (100%)	
Others	8	1 (12.5%)	7 (87.5%)	

IQR, interquartile range.

## Data Availability

All relevant data are within the manuscript.

## Supplementary Materials

The following supporting information can be downloaded at: https://www.mdpi.com/article/10.3390/diagnostics14212392/s1, Table S1: Detailed clinical summary.
